# ACMG-Recommended Actionable Secondary Findings from 1600 Clinical Exomes in the South Marmara Region in Turkiye

**DOI:** 10.3390/ijms27031491

**Published:** 2026-02-03

**Authors:** Sehime Gulsun Temel, Mustafa Samet Pir, Cuneyd Yavas, Feride I. Sahin, Sebnem Ozemri Sag, Yunus Kasim Terzi

**Affiliations:** 1 Department of Medical Genetics, Faculty of Medicine, Bursa Uludag University, Bursa 16059, Türkiye; sehime@uludag.edu.tr; 2 Department of Translational Medicine, Institute of Health Sciences, Bursa Uludag University, Bursa 16059, Türkiye; 3 Department of Biostatistics and Bioinformatics, Institute of Health Sciences, Acibadem University, Istanbul 34638, Türkiye; mustafapir29@gmail.com; 4 Department of Molecular Biology and Genetics, Faculty of Engineering and Natural Sciences, Biruni University, Istanbul 34015, Türkiye; cyavas@biruni.edu.tr; 5 Department of Medical Genetics, Faculty of Medicine, Baskent University, Ankara 06790, Türkiye; feridesahin@hotmail.com

**Keywords:** clinical exome sequence, secondary findings, variant, population frequency

## Abstract

In genetic disease assessment centers, DNA sequencing can produce results irrelevant to the genetic examination’s purpose. The American College of Medical Genetics and Genomics (ACMG) recommends evaluating and reporting 81 genes discovered using clinical genomic sequencing. While population studies on large cohorts can provide statistics on the prevalence of secondary findings (SFs), no studies have been published yet on large cohorts in Turkiye. We investigated ACMG SF by evaluating clinical exome sequencing data in 1600 individuals from different regions in Turkiye. We detected SF variants reported in ClinVar in 86 individuals (5.375%). Of the SFs, 30% were cardiovascular, 26% were cancer, 16% were neonatal metabolic disorders, and 28% were variants associated with various genetic diseases. In addition, we identified 212 different variants in 226 individuals and 45 different genes, which were not reported in ClinVar. When our results are compared with the Turkish National Genome and Bioinformatics Project database and studies in the literature, the studies vary in terms of participant characteristics, sequencing techniques, and versions of the ACMG SF list. Our findings highlight the importance of expanding and tailoring SF reporting guidelines in populations with high consanguinity and limited cohort-based data.

## 1. Introduction

Advancements in sequencing technology and decreasing costs have accelerated the identification of genetic diseases in both academic and clinical settings. Genetic sequencing technologies, including whole genome sequencing (WGS), whole exome sequencing (WES), and clinical exome sequencing (CES), yield vast amounts of data on the human genome and associated disorders. This accelerates and simplifies the search for diseases characterized by significant genetic diversity or ambiguous findings. Next-generation sequencing (NGS) may also reveal unexpected secondary findings (SF) that are unrelated to the primary indication for testing [1]. Secondary findings are medically actionable variants that may indicate an increased risk for diseases beyond the patient’s primary diagnosis [1]. They are interpreted as genetic findings identified during genomic testing that are not associated with the participant’s clinical presentation or the original indication for testing [1]. There is still no consensus on whether these secondary findings should be reported. Some argue for publishing secondary findings where there is evidence of a pathogenic (P) or likely pathogenic (LP) variant that may cause an illness that might benefit from early care [2]. Secondary findings enable timely medical interventions, and personalized preventive strategies that can substantially improve patient outcomes and reduce long-term healthcare costs. Moreover, secondary findings contribute positively by expanding our understanding of the clinical significance of genetic variants, improving risk stratification, and guiding preventive healthcare strategies at both the individual and population levels. Furthermore, these technologies can reveal variants in genes unrelated to the original condition, necessitating genetic testing, which may result in life-threatening and/or preventive disorders. Genetic diseases may affect all family members; hence, publishing variants of most non-legal probands might lead to ethical quandaries [3].

The American College of Medical Genetics and Genomics (ACMG) and the Association for Molecular Pathology (AMP) recommend clinical sequencing laboratories to disclose P and LP variants irrespective of patient age, even if they are not directly linked to the primary indication for genetic testing. In 2013, ACMG issued guidelines outlining the reporting of incidental findings discovered during clinical sequencing, particularly those associated with Mendelian disorders considered clinically secondary findings [2]. ACMG established recommendations for reporting genomic data on 56 genes linked with clinically relevant and severe symptoms. The genes in this guideline were revised to 59, 73, 78, and 81 in August 2023, with the advice to report P and LP variants. These genes are associated with high-penetrance variants that lead to disease. Individuals carrying P and LP variants in these genes are at high risk of developing diseases [4] that can be treated or prevented to reduce future morbidity and/or mortality [5,6,7,8]. While the prevalence and epidemiology of medically secondary findings can be derived from large population studies, there is a lack of data on genetic variants common in the Turkish, Arab, and Middle Eastern populations where consanguineous marriages are common [9,10,11].

One of the major issues with implementing the ACMG SF standards is that, to date, experts’ opinion on including these gene–disease combinations in the ACMG SF list is not based on the testing of larger patient groups. In this study, we aimed to identify and thoroughly analyze secondary findings within the 81 ACMG-recommended genes using clinical exome sequencing data from 1600 Turkish patients. This is a systematic study to identify clinically relevant findings to enable clinical prediction for an individual’s health, enabling new approaches to preventing genetic diseases and preventive medicine. To our knowledge, this study is the largest and most comprehensive analysis to estimate variants of medically secondary findings in Turkiye. In addition, the results of this study will pioneer future studies on ACMG 81 secondary findings (ACMG81 SF) in the Turkish population, highlighting the importance of large-cohort analyses to assess prevalence, clinical relevance, and population-specific preventive genomic medicine.

## 2. Results

### 2.1. Cohort Characteristics

**Figure 1:**
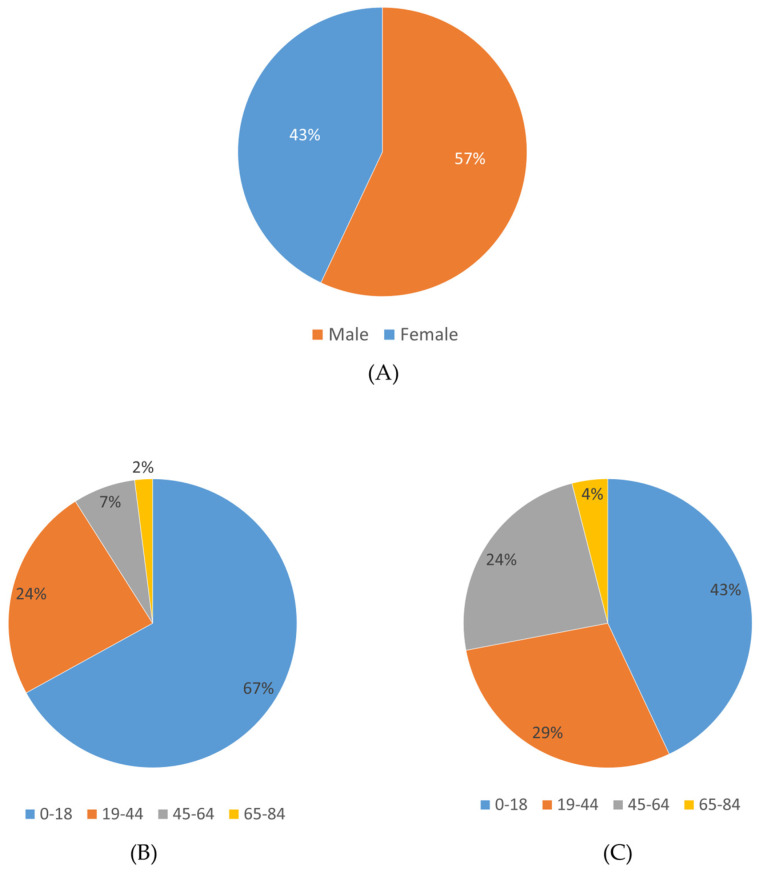
Gender and age distribution of the study cohort. (**A**) Proportion of male and female patients. (**B**) Age distribution among male patients. (**C**) Age distribution among female patients.

**Figure 2:**
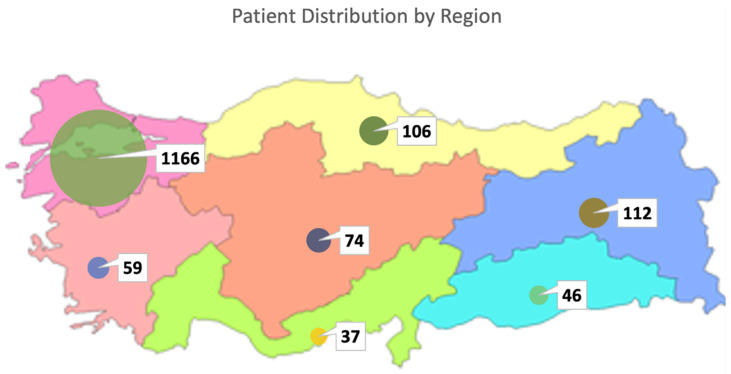
Regional distribution of patients in Turkiye.

In this study, clinical exome sequencing data from 1600 patients were analyzed, of whom 57% were female and 43% were male (Figure 1A). The mean age of the cohort was 24.12 ± 12 years. Age distribution analysis revealed that the majority of patients were within the 0–18 age group (Figure 1B,C). The largest proportion of patients was from the Marmara region (1166 patients, 72.8%), followed by the Eastern Anatolia region (112 patients, 7%) and the Black Sea region (106 patients, 6.6%), with the majority self-identifying as Turkish (Figure 2) (Table 1).

### 2.2. Frequency and Distribution of ACMG Secondary Findings

Variants in one of the 81 ACMG-recommended genes were identified in 45.5% of patients (729/1600). Among these, secondary findings classified as pathogenic/likely pathogenic in ClinVar were detected in 86 patients (5.375%) (Figure S1). The most frequently affected genes were *MUTYH* (8 patients), *ATP7B* (15 patients), *BTD* and *GAA* (in 7 patients each), *MYH7* (6 patients), *APOB* (4 patients), and various other genes (Figure S1).

Overall, 56 different ClinVar-reported P/LP variants were identified (Figure S2). These included 36 missense variants, 1 located in the 5′UTR, 2 frameshift variants, 1 amino acid deletion, 1 intronic variant, 12 nonsense variants, and 3 splice site variants.

The overall frequency of reportable secondary findings in the 81 ACMG-recommended genes was 5.375% (86/1600), while no reportable variants were observed in 94.625% of patients (1514/1600). At the gene level, 25 of the 81 ACMG-recommended genes (30.8%) harbored at least one reportable variant, whereas no variants were detected in the remaining 56 genes (69.2%).

Among the 81 ACMG-recommended genes, the most frequently observed variant was *MUTYH* c.842C>T, followed by *MUTYH* c.1145G>A, *ATP7B* c.3207C>A, *TTN* c.35876-2A>G, and others. The *MUTYH* c.842C>T variant, classified as P/LP, was detected in nine patients, corresponding to a frequency of 10.3%. The allele frequencies of variants detected in ACMG secondary finding genes are shown in Figures S3 and S4.

**Figure 3:**
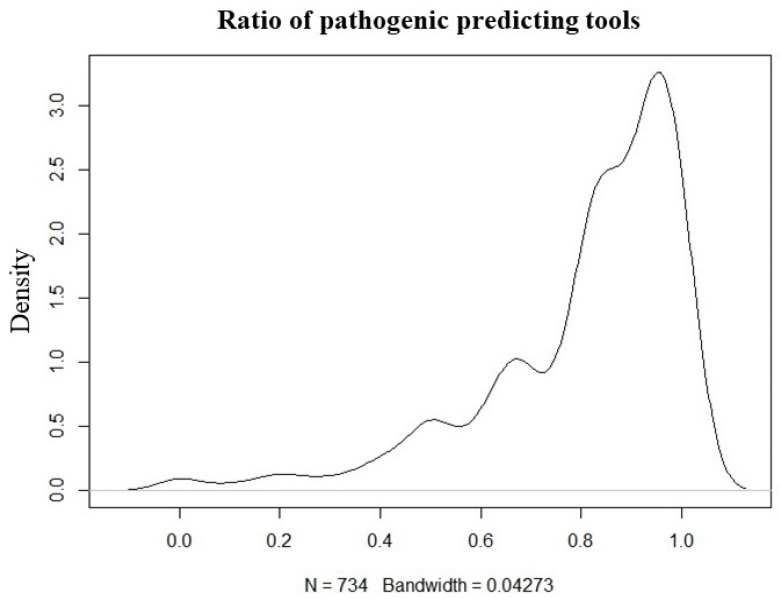
Ratio plot of the tools used to predict the pathogenicity of variants for unreported pathogenicity in ClinVar (N = variants).

Except for *HFE* gene variants, variants with no information on pathogenicity in ClinVar were predicted using InterVar and ANNOVAR tools, and 212 variants predicted to be pathogenic in 45 different genes were detected in 226 patients. The variant number and ClinVar report information findings of the study are shown in Figure 3.

### 2.3. Variants Reported in ClinVar

**Figure 4:**
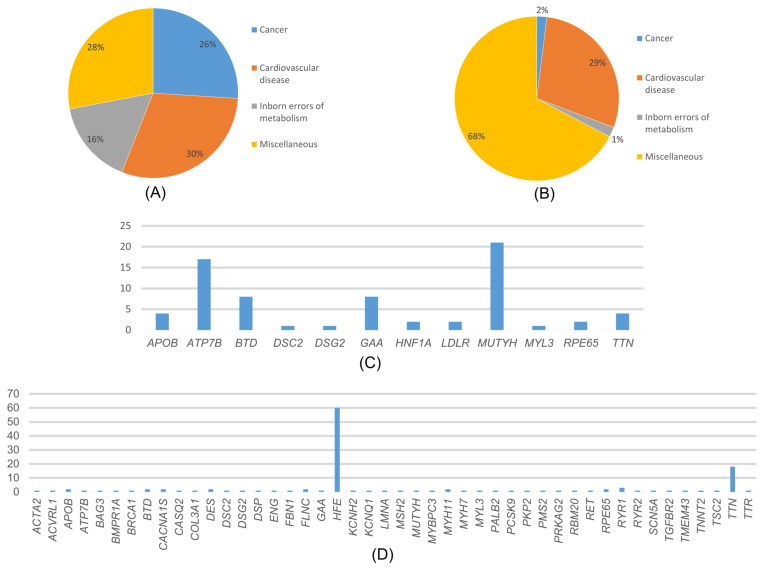
(**A**) Distribution of the variants detected in our study and reported in ClinVar according to diseases. (**B**) Distribution of variants detected in our study that are not reported in ClinVar, according to disease categories. (**C**) Distribution of the variants detected in our study and reported in ClinVar according to genes. (**D**) Distribution of variants detected in our study that are not reported in ClinVar, according to genes.

Among the variants reported as P/LP in ClinVar, the most common disease-associated genes were those related to cardiovascular diseases (30%), cancer (26%), neonatal metabolic disorders (16%), and various other conditions such as diabetes, neurological diseases, and eye diseases (28%) (Figure 4A). In the distribution of cancer-associated variants by genes, the *MUTYH* gene accounted for 78.26% of variants, while *VHL*, *TSC2*, *SDHB*, *PALB2*, and *BRCA1* each accounted for 4.35% (Figure 4C).

In the *MUTYH* gene, variants were detected in 18 individuals (1.125%), and the most common variant, c.842C>T variant, was detected in 9 individuals. In the distribution of variants associated with cardiovascular diseases, *MYH7* accounted for 23.08%, *APOB* for 15.38%, *MYBPC3* and *TTN* for 11.54%, and the rest is attributed to other genes. Among these genes, the most detected variants were *APOB* 13151T>C, *MYH7* c.2134C>T, and *TTN* c.35876-A>G. Among the genes detected in cardiovascular diseases, only the *MYBPC3* gene c.3331-1G>A variant was homozygous. All other variants were heterozygous. The distribution of variants associated with congenital metabolic disorders by genes was 50% *BTD* and *GAA* genes. The most frequently detected variant among these genes was *GAA* c.-45T>A. In addition, all the detected variants were heterozygous. In the distribution of variants associated with various genetic diseases by genes, 62.5% of variants were found in the *ATP7B* gene, 20.83% in the *RYR1* gene, and 8.33% in the *RPE65* and *HNF1A* genes each. Among the variants reported as P/LP in ClinVar, the *ATP7B* gene was the most common among genes associated with various genetic diseases. Among these genes, the most frequently detected variant was *ATP7B* c.3207C>A.

ClinVar P/LP variants were clearly distinguished from variants not reported in ClinVar, with not reported in ClinVar variants representing computationally predicted or currently unclassified findings rather than established ACMG-reportable secondary findings, particularly in gene- and disease-level frequency analyses.

### 2.4. Variants Not Reported in ClinVar

Variants which were detected in 547 patients (34.187%) were not reported as P/LP in ClinVar but were predicted to be pathogenic by pathogenicity prediction tools such as ANNOVAR and InterVar. These variants were associated with cardiovascular diseases (29%), cancer (2%), congenital metabolic disorders (1%), and other conditions (68%) (Figure 4B). In our study, we found that, among the cancer-related genes with no information about pathogenicity in ClinVar, *MSH2*, *BRCA1*, and *TSC2* had the highest number of variants at 18.8% each, followed by *PALB2* at 9.09%, and other genes. Among these genes, the most detected variant was *MSH2* c.2670G>T, and all variants detected were heterozygous. The distribution of variants associated with cardiovascular diseases by genes is as follows: 61.62% *TTN*, 4.32% *RYR2*, 3.24% *SCN5A,* and the rest are distributed among other genes (Figure 4D). The most frequently detected variant among these genes was *TTN* c.63158G>A. Among these variants, the *KCNQ1* gene has the c.461G>A and c.1546G>A variants, while the *TTN* gene has the c.22229G>A and c.5023C>T variants, all of which were homozygous. The rest of the variants were heterozygous. The distribution of variants associated with congenital metabolic disorders by genes was 66.67% *BTD* and 33.3% *GAA* genes. All detected variants were heterozygous. The most frequently detected variant among these genes was *BTD* c.104G>C. The distribution of variants associated with other phenotypes by genes was as follows: 89.63% *HFE*, 5.07% *RYR1*, 1.84% *CACNA1S*, 1.15% *RPE65*, 0.92% *ACVRL1* and *ATP7B*, 0.23% *TTR* and 0.23% *ENG* genes.

Among the genes recommended in the ACMG81 SF guideline, only the p.(Cys282Tyr) variant of the *HFE* gene is recommended for reporting [5]. In our study, *HFE* gene variants were detected in 385 patients. The variants in this gene were as follows: p.(His63Asp) was homozygous in 23 of 361 patients and heterozygous in 341 patients, p.(Cys282Tyr) was homozygous in 1 of 16 patients and heterozygous in 15 patients, and p.(Ser65Cys) was heterozygous in all nine patients.

### 2.5. Cardiovascular Disease (CVD)–Associated Genes

As a result of our study, when CVD-associated genes were analyzed; *APOB*, *MYBPC3*, *MYH7,* and *TTN* were found to be the genes with the most variants. Some of these variants have been reported as pathogenic/likely pathogenic in ClinVar. In *APOB* gene, the c.13151T>C variant reported in ClinVar was detected in one patient (0.0625%) and the c.7103A>G, c.11840G>T, c.1931C>A, c.10315A>T, and c.6568G>T variants not reported in ClinVar were detected in six patients (0.375%). In *MYBPC3* gene, the c.1483C>T, c.2827C>T, and c.3331-1G>A variants reported in ClinVar were detected in three individuals (0.1875%) and the c.2344A>T variant not reported in ClinVar was detected in one individual (0.0625%). *MYH7* gene variants c.2134C>T, c.2606G>A, c.2146G>A, c.488A>C, and c.1640C>T reported in ClinVar were detected in six individuals (0.375%) and the c.4356C>G variant not reported in ClinVar was detected in one individual (0.0625%). In the *TTN* gene, two ClinVar-reported variants (c.35876-2A>G and c.54278_54279delCT) were detected in three individuals (0.1875%). In addition, 93 distinct variants not reported in ClinVar were identified in 111 individuals (6.9375%) (Table 2).

### 2.6. Cancer-Associated Genes

In our study, *BRCA1*, *MUTYH*, *SDHB*, and *VHL* variants were detected. In the *BRCA1* gene, the c.3770_3771delAG variant reported in ClinVar was detected in one individual (0.0625%) and the c.856G>C and c.864C>A variants not reported in ClinVar were detected in two individuals (0.125%). In the *MUTYH* gene, the c.1129C>T, c.842C>T, c.1145G>A, c.692G>A, and c.270C>A variants reported in ClinVar were detected in 18 individuals (1.125%) and the c.1383G>T variant not reported in ClinVar was detected in one individual (0.0625%). The c.143A>T variant reported in ClinVar in the *SDHB* gene was detected in one individual (0.0625%). The c.499C>T variant of the *VHL* gene reported in ClinVar was detected in one individual (0.0625%).

### 2.7. Neonatal Metabolic Disorders

*BTD* gene variants, frequently associated with neonatal metabolic disorders, were identified in our cohort. The variants c.1368A>C, c.1613G>A, c.469C>T, c.1369G>A, c.470G>A, and c.1595C>T reported in ClinVar were detected in seven individuals (0.4375%) in our study. The variants c.749T>A, c.1450T>C, c.793C>T, and c.104G>C, which were not reported in ClinVar, were detected in eight (0.5%) individuals. The c.470G>A variant was reported in the Turkish Genome Project Data Sharing Portal (TGPDSP) and its frequency was reported as 0.001. In our study, the frequency of this variant was found as 0.001875. Importantly, these variants were not reported in the previous study conducted on Turkish patients by Ates et al. [12] and were reported for the first time in the Turkish population.

## 3. Discussion

A comprehensive CES dataset in this investigation prompted an examination of the prevalence of 81 genes (ACMG SF v3.2) within the Turkish population. This demographic has largely eluded characterization due to the absence of extensive population-based genomic resources in Turkiye. We analyzed 1600 CES samples harboring variants categorized as pathogenic or likely pathogenic (P/LP) according to ClinVar records, with further validation through ANNOVAR and InterVar pathogenicity prediction tools. Our analysis unveiled 56 P and LP variants documented in ClinVar across 25 ACMG genes, observed in 86 unrelated individuals. Notably, 5.375% of the cohort exhibited a P or LP variant within the 81 ACMG genes analyzed. Among these, 5.375% represented directly reportable variants. Additionally, we identified 212 predicted P/LP variants not reported in ClinVar (not-CV), spanning 45 ACMG genes across 226 patients, except the *HFE* gene, which was singled out through pathogenicity prediction tools. Variants without pathogenicity information in ClinVar other than *HFE* gene variants were predicted with InterVar and ANNOVAR tools, and 212 different variants predicted to be pathogenic in 45 different genes were found in 226 patients. When *HFE* gene variants were added to this, variants were found in 547 patients (34.187%). More evidence is needed to fully characterize these predicted pathogenic variants as pathogenic. First, family segregation of the identified variants must be performed. In addition, a literature review and functional studies, if possible, will increase the evidence. Variants that may be considered to be pathogenic after these studies can be included in reportable patients.

Previous studies reported ACMG SF rates ranging from 0.6% to 6.6%. Aloraini et al. found that the total rate of SFs in the Saudi population was greater than 8% [13,14,15]. Studies such as the MyCode Community Health Initiative have shown a significant increase in the positive screening rate of 19–24% when including ACMG SF v3.0 genes as opposed to the earlier v2.0 genes (from 2.1–2.6% to 2.6–3.1%) [16]. The number of participants in the SF studies varied from less than 200, 161 [17], 196 [14], or less than 300, 280 [18] to 1000, 6240 [19] and 21,915 [20]. Research with a high sample size could bring about ethical or budgetary challenges; likewise, research with limited sample sizes may prohibit findings from being generalized to the population level. The highest reported SF rates (6.6% and 6.1%) were observed in studies with relatively small cohorts of 196 and 280 participants, respectively [8,14,17,18,19].

Most SF research has been conducted in local communities, such as Saudi Arabia, China, Qatar, Lebanon, Korea, Taiwan, the Netherlands, Singapore, and Thailand. There were more representative studies with reported ethnic groups, such as the 1000 Genomes Project, which included 14 different populations: Hispanic or Latino, black or African American, Asian, American Indian, Alaska Native or Pacific Islander, white, East Asian, European, and African American [13,14,15,17,18,19,21,22,23,24]. There are few studies that have had a large number of Turkish participants. Ates et al. conducted a CES investigation with 622 individuals [12].

Depending on the time of publication, the versions used in the studies vary because the ACMG gene list is expanding. As far as we know, there are ACMG SF v3.0 and v3.1 studies. However, there are not many v3.2 studies. Especially in the Turkish population, our study is the first with the most recent ACMG version and number of participants. In a study conducted to determine the frequency of P/LP variants of ACMG SF v2.0, the frequency of SF was reported as 2.85% [25]. Studies using the ACMG SF v3.2 list have not yet been conducted. Considering the slight difference in the number of genes between ACMG lists v1.0 and v2.0 (56 genes to 59 genes), a significant increase in SFs was not expected. In this case, it is important to bring new studies to the literature to answer whether there will be a significant increase or decrease between the v3.1 and v3.2 (79 genes to 81 genes) versions. Increases in results have been observed with the increase in versions. In our study, the increases and decreases in the old version studies with a similar number of participants were shown, and it is the first study conducted within the Turkish population.

Disease-causing copy number variants (CNVs) are responsible for 5–9% of genetic diseases [26,27,28]. CES can identify CNVs, but its specificity and sensitivity are limited to long deletion and duplication changes. Although most studies on SF have utilized CES, WES, and WGS, CNVs have not been included in these analyses. In our study, CNVs could not be evaluated because it was not possible to retrieve CNV data anonymously from the SOPHIA DDM platform. Therefore, large deletion and insertion variants in genes such as *BRCA1*, *BRCA2,* and *SMN1* were excluded from the filtering, and these variants could not be detected. This is one of the limiting factors of our study.

In previous ACMG SF study findings, CVD and cancer were the most prevalent disease categories. The prevalence of CVD ranged from 11.7% [18] to 54.5% [23] whereas the prevalence of cancer ranged from 16.7% [24] to 75.0% [25]. In our study, the high prevalence of CVD (30%) can be partly attributed to the varied ACMG versions employed. We used ACMG version 3.2 and found two different *TTN* variants in three individuals (c.35876-2A>G and c.54278_54279delCT). It is worth noting that previous editions of the ACMG SF list did not include *TTN* cardiomyopathy, and its inclusion significantly raised the percentage of CVD while decreasing the percentage of other diseases.

Although the update from ACMG v3.1 to v3.2 also introduced additional CVD-associated genes, including *CALM1*, *CALM2*, and *CALM3*, no pathogenic or likely pathogenic variants in these genes were detected in our cohort. Therefore, the observed increase in CVD prevalence in this study reflects cohort-specific findings. The genes found in ACMG81 SF were cancer (26%), neonatal metabolic disorders (16%), and various genetic diseases. Analysis of variants not reported in ClinVar by disease category showed that most were associated with various genetic disorders (68%), followed by cardiovascular diseases (29%), cancer (2%), and neonatal metabolic disorders (1%).

In our study, *MYH7*, *APOB*, *MYBPC3*, *TTN*, and *LDLR* were the most frequently observed cardiovascular disease genes, and *MUTYH* was the most frequently observed cancer gene among ACMG SFs. *TTN* and *HFE* were most frequent among not-CV SF (Figure 4), and *MSH2*, *BRCA1*, and *TSC2* were the most frequent cancer genes among not-CV SF. These findings suggest that variants associated with cardiovascular disease and cancer may be relatively frequent in the Turkish population; however, this observation should be interpreted with caution, particularly for variants identified through prediction-based approaches.

In a study of a Turkish cohort, nine individuals (1.44%) had variants in genes related to cardiovascular disease, and seven individuals (1.12%) had variants in genes related to cancer, whether reported or not in ClinVar [12]. The prevalence of these variants was higher in our study compared to previous findings. According to researchers, individuals carry a disease allele in 0.40% to 3.1% of genetic diseases with AR mode of inheritance from the ACMG SF list v2.0 [15,17,19]. Despite an increase in the number of AR diseases on the ACMG81 SF list, our cohort had a higher carrier rate (3.8%). This is attributable to the fact that our cohort had fewer ACMG81 SF results than the research described above.

In the TGPDSP database, the reported frequencies of *HFE* c.193A>T, c.845G>A, and c.187C>G variants were 0.006, 0.004, and 0.093, respectively. According to the results of our study, the frequencies of these variants were 0.0028125, 0.0053125, and 0.1209375. Compared with the TGPDSP database, the frequencies of the *HFE* c.193A>T and c.845G>A variants were lower in our cohort, whereas the frequency of the c.187C>G variant was higher. Similarly, the TGPDSP database reported frequencies of 0.004 for the *MUTYH* c.842C>T variant, 0.003 for the *RYR1* c.11414A>G variant, and 0.003 and 0.002 for the *TTN* c.72530A>C and c.92282C>G variants, respectively. Similarly, the TGPDSP database reported the frequencies of the *MUTYH* c.842C>T, *RYR1* c.11414A>G, *TTN* c.72530A>C, and c.92282C>G variants to be 0.004, 0.003, 0.003, and 0.002, respectively. In our study, these frequencies were found as 0.005625, 0.00125, 0.000625, and 0.000625. In this case, it was seen that our study was low in terms of these variants. The frequencies of variants detected in our study but not reported in the TGPDSP database are provided in Supplementary Table S1.

Conducting CES studies to detect ACMG 3.2 SF variants offers advantages in terms of speed and cost-effectiveness compared to WES and WGS. Our findings support the utility of CES as a cost-effective approach for identifying ACMG secondary findings; however, conclusions regarding epidemiological significance and clinical impact should be interpreted cautiously, particularly for variants identified through in silico prediction. Prediction-based variants primarily serve a hypothesis-generating role and highlight candidates for future validation rather than definitive clinical interpretation. The observed frequency of ACMG SF variants (5.375%) falls within the reported range in pertinent literature. The elevated prevalence of CVD among ACMG SFs in our study (30%) may be attributed partly to the inclusion of CVD-related genes, such as *TTN* and *DES*, in the ACMG v3.0, v3.1, and v3.2 SF lists. Furthermore, our investigation using CES v2 in Turkiye reported a frequency of 2.1%, which increased with subsequent version updates and greater participant numbers. These findings may be relevant to Turkiye’s epidemiological landscape, characterized by a high prevalence of consanguineous marriages. Our results could inform advancements in genomic medicine practices, particularly in countries with pronounced consanguinity rates, advocating for the evaluation of variant pathogenicity using predictive algorithms for variants not reported in ClinVar.

Importantly, our study was conducted prior to the initiation of the TGPDSP, which aims to establish a comprehensive genomic reference resource for the Turkish population. In the absence of such a national-scale dataset, our findings provide one of the first large-scale evaluations of ACMG v3.2 secondary findings in Turkiye, offering valuable baseline data for the interpretation of genetic variation. As the TGPDSP progresses, the results of our study may serve as a comparative reference, supporting the refinement of population-specific variant classification and contributing to the integration of genomic medicine into national healthcare systems.

## 4. Materials and Methods

### 4.1. Gene Selection and ACMG v3.2 Secondary Findings

The genes under examination comprise the 81 SF genes, in accordance with the guidelines set forth by ACMG v3.2 secondary findings. This list includes 81 medically actionable genes selected based on disease severity, penetrance, and clinical actionability. These guidelines mandate their inclusion in the results of all clinical genomic sequencing laboratories, irrespective of their relevance to the initial medical query. This directive stems from their correlation with highly penetrant variants manifesting disease phenotypes [5].

### 4.2. Clinical Exome Sequencing and Data Processing

Clinical Exome Solution v2 kit (SOPHiA Genetics, Boston, MA, USA) was used for exome enrichment following the manufacturer’s protocol. This capture-based target enrichment kit covers 4493 genes with known inherited disease-causing variants. Paired-end sequencing was performed on an Illumina NextSeq 500 system (Illumina Inc., San Diego, CA, USA) with a read length of 150 × 2. Base calling and image analysis were performed using Illumina’s Real-Time Analysis software v2.11.3, and BCL files were converted to FASTQ format using the bcl2fastq package v1.8.4. ACMG SF genes were included to evaluate secondary findings; after quality control, variants were analyzed by ANNOVAR [29] using ExAC [30], gnomAD [31], 1000 Genomes [32], and ClinVar [33] databases.

### 4.3. Variant Filtering and Classification Strategy

**Figure 5:**
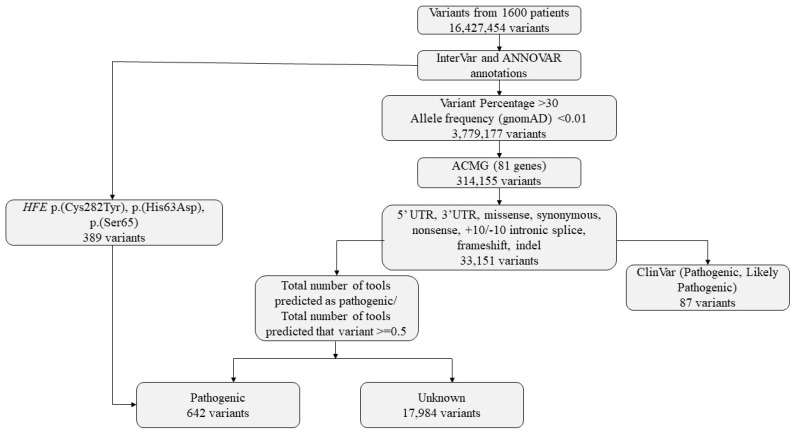
Variants identified in the study and bioinformatics flowchart.

Variants obtained from CES of 1600 individuals were subjected to a multi-step filtering and classification pipeline (Figure 5). Raw variant calls were first annotated using ANNOVAR [29] and InterVar [34]. Initial filtering excluded variants with low allele representation, retaining only those with allele balances greater than 30% and minor allele frequency (MAF) < 0.01 in the gnomAD database. Variants meeting these criteria were further limited to the 81 genes identified as actionable in the latest ACMG secondary findings list (v3.2). Only variants located in regions of potential functional relevance—such as 5′UTR, 3′UTR, missense, synonymous, nonsense, frameshift, indels, and ±10 bp intronic splice sites—were considered. In analyzing variants from ACMG SF genes, those classified as P/LP in ClinVar were prioritized. Only coding region and canonical splice site variants were included. Single nucleotide variants (SNVs) and indels were classified according to ACMG/AMP guidelines. Due to the limitations in detecting copy number variants (CNVs), CNV analysis was not included in this study.

### 4.4. Variant Annotation and Pathogenicity Assessment

Variants were then categorized based on ClinVar classification. Those reported as pathogenic, likely pathogenic, or pathogenic/likely pathogenic were marked accordingly. For variants without ClinVar classification, in silico pathogenicity predictions were obtained using InterVar (v2.2.2, 27 July 2021) together with multiple prediction scores annotated via ANNOVAR (release 16 April 2018), which includes 22 prediction tools (included in hg19_dbnsfp42a database). A pathogenicity ratio was calculated as the proportion of available prediction tools indicating a deleterious or pathogenic effect relative to the total number of tools providing a prediction for that variant. (Figure 3). A threshold of ≥0.5 was used to define candidate pathogenic variants for prioritization. This threshold was selected empirically based on internal benchmarking using ClinVar-annotated variants, where variants predicted as pathogenic by at least 50% of predictors showed substantial concordance with ClinVar pathogenic/likely pathogenic classifications.

Three *HFE* gene variants (p.(Cys282Tyr), p.(His63Asp), p.(Ser65Cys)), known to be associated with hereditary hemochromatosis, were included due to their clinical relevance regardless of ClinVar annotation. This tiered approach, integrating clinical databases, population frequency data, functional annotations, and in silico predictions, was designed to support systematic variant prioritization and downstream evaluation.

## Figures and Tables

**Table 1:** Number of patients included in the study according to demographic characteristics of the patients.

Region	Patient Count	ClinVar Reporter Variant	Not-Reporter in ClinVar
Aegean Region	59	4	18
Black Sea Region	106	9	49
Central Anatolia Region	74	2	33
Eastern Anatolia Region	112	4	19
Marmara Region	1166	64	415
Mediterranean Region	37	-	14
Southeastern Anatolia Region	46	3	14

In this table, patients carrying *HFE* gene c.845G>A, c.193A>T, and c.187C>G variants were not included.

**Table 2:** Number of patients and inheritance patterns of pathogenic/likely pathogenic reported and unreported variants detected in ClinVar (Number of patients = n).

Gene	Hereditary Pattern	Reported in ClinVar (n)	Not Reported in ClinVar (n)	Variants Predicted to Be Pathogenic (n)
*ACVRL1*	AD	-	31	4
*ACTA2*	AD	-	5	1
*APOB*	AD-AR	4	582	6
*ATP7B*	AR	15	58	4
*BAG3*	AD	-	12	3
*BMPR1A*	AD	-	305	1
*BRCA1*	AD	1	164	2
*BTD*	AR	7	105	8
*CACNA1S*	AD	-	81	8
*CASQ2*	AR	-	44	1
*COL3A1*	AD-AR	-	72	2
*DES*	AD-AR	-	15	5
*DSC2*	AD-AR	1	53	2
*DSG2*	AD-AR	1	14	1
*DSP*	AD-AR	-	142	4
*ENG*	AD	-	998	1
*FBN1*	AD	1	278	2
*FLNC*	AD	-	169	5
*GAA*	AR	7	96	4
*HFE*	AR	-	12	-
*HNF1A*	AD-AR	2	28	-
*KCNH2*	AD	-	642	1
*KCNQ1*	AD-AR	1	468	2
*LDLR*	AD-AR	2	531	-
*LMNA*	AD-AR	1	69	1
*MSH2*	AD-AR	-	4	2
*MUTYH*	AR	18	4	1
*MYBPC3*	AD-AR	3	411	1
*MYH11*	AD-AR	-	71	5
*MYH7*	AD-AR	5	39	1
*MYL3*	AD-AR	1	7	1
*PALB2*	AD	1	9	1
*PCSK9*	AD	-	426	1
*PKP2*	AD	-	47	4
*PMS2*	AR	-	251	1
*PRKAG2*	AD	-	33	1
*RBM20*	AD	-	17	3
*RET*	AD	-	1008	1
*RPE65*	AD-AR	2	14	5
*RYR1*	AD-AR	5	323	22
*RYR2*	AD	-	525	8
*SCN5A*	AD-AR	1	1005	6
*SDHB*	AD-AR	1	7	-
*TGFBR2*	AD	1	6	1
*TMEM43*	AD	-	11	1
*TNNT2*	AD	-	21	2
*TSC2*	AD	1	128	2
*TTN*	AD-AR	3	680	111
*TTR*	AD	-	5	1
*VHL*	AD-AR	1	303	-

AD: Autosomal dominant, AR: Autosomal recessive.

## Data Availability

Data are available on request from the corresponding author. The data are not publicly available due to privacy or ethical restrictions.

## Supplementary Materials

The following supporting information can be downloaded at: https://www.mdpi.com/article/10.3390/ijms27031491/s1.
