# Supplementary material for: ACMG-Recommended Actionable Secondary Findings from 1600 Clinical Exomes in the South Marmara Region in Turkiye
Source: Int J Mol Sci. 2026 Feb 3;27(3):1491. doi: 10.3390/ijms27031491 (PMC12898675; doi:10.3390/ijms27031491)
Supplement: Supplementary file 1 [file ijms-27-01491-s001.zip › ijms-4086442-supplementary.pdf]

Table S1. Comparison of the variants identified in the study with international and national databases

Gene	Chr	Locus (hg19)	Transkript	cDNA	Protein	dbSNP ID	ClinVar Accession	TGPDSPTF	Freq	AF	GnomAd
ACTA2	10	90695114	NM_001141945	c.1000C>A	p.(Pro334Thr)	NA	NA	NA	0,000625	0,000313	NA
ACVRL1	12	52309077	NM_000020	c.841G>C	p.(Glu281Gln)	NA	NA	NA	0,00125	0,000625	NA
ACVRL1	12	52309149	NM_000020	c.913T>A	p.(Ser305Thr)	NA	NA	NA	0,000625	0,000313	NA
ACVRL1	12	52307552	NM_000020	c.523G>C	p.(Gly175Arg)	NA	NA	NA	0,000625	0,000313	NA
APOB	2	21225143	NM_000384	c.13151T>C	p.(Leu4384Pro)	rs1339117465	VCV000440508.1	NA	0,001875	0,000938	NA
APOB	2	21232182	NM_000384	c.7558C>T	p.(Arg2520*)	rs746483297	VCV001983784.2	NA	0,000625	0,000313	NA
APOB	2	21227496	NM_000384	c.11840G>T	p.(Gly3947Val)	NA	NA	NA	0,000625	0,000313	NA
APOB	2	21232637	NM_000384	c.7103A>G	p.(Tyr2368Cys)	NA	NA	NA	0,00125	0,000625	NA
APOB	2	21250836	NM_000384	c.1931C>A	p.(Ser644Tyr)	NA	NA	NA	0,000625	0,000313	NA
APOB	2	21229425	NM_000384	c.10315A>T	p.(Asn3439Tyr)	NA	NA	NA	0,000625	0,000313	NA
APOB	2	21233172	NM_000384	c.6568G>T	p.(Asp2190Tyr)	NA	NA	NA	0,000625	0,000313	NA
ATP7B	13	52511697	NM_000053	c.3818C>T	p.(Pro1273Leu)	rs758355520	VCV000189139.41	NA	0,000625	0,000313	NA
ATP7B	13	52531716	NM_000053	c.2383C>T	p.(Leu795Phe)	rs751710854	VCV000188814.24	NA	0,000625	0,000313	NA
ATP7B	13	52523859	NM_000053	c.2804C>T	p.Thr935Met	rs750019452	VCV000188713.38	NA	0,00125	0,000625	2,00E-04
ATP7B	13	52535995	NM_000053	c.1924G>C	p.(Asp642His)	rs72552285	VCV000526655.12	NA	0,000625	0,000313	NA
ATP7B	13	52523836	NM_000053	c.2827G>A	p.(Gly943Ser)	rs28942076	VCV000558661.2	NA	0,000625	0,000313	NA
ATP7B	13	52513227	NM_000053	c.3659C>T	p.(Thr1220Met)	rs193922107	VCV000035725.23	NA	0,000625	0,000313	NA
ATP7B	13	52532674	NM_000053	c.2128G>A	p.(Gly710Ser)	rs137853285	VCV000156281.57	NA	0,00125	0,000625	NA
ATP7B	13	52518281	NM_000053	c.3207C>A	p.(His1069Gln)	rs76151636	VCV000003848.88	NA	0,00375	0,001875	0,001
ATP7B	13	52511519	NM_000053	c.3914T>A	p.(Leu1305Gln)	NA	NA	NA	0,000625	0,000313	NA
ATP7B	13	52539088	NM_000053	c.1789G>A	p.(Val597Ile)	NA	NA	NA	0,000625	0,000313	NA
ATP7B	13	52542698	NM_000053	c.1589T>C	p.(Ile530Thr)	NA	NA	NA	0,000625	0,000313	NA
ATP7B	13	52539050	NM_000053	c.1827C>A	p.(Asp609Glu)	NA	NA	NA	0,000625	0,000313	NA
BAG3	10	121436441	NM_004281	c.1375A>G	p.(Thr459Ala)	NA	NA	NA	0,000625	0,000313	NA
BAG3	10	121436597	NM_004281	c.1531C>A	p.(Gln511Lys)	NA	NA	NA	0,000625	0,000313	NA
BAG3	10	121429391	NM_004281	c.209C>G	p.(Ser70Cys)	NA	NA	NA	0,000625	0,000313	NA
BMPR1A	10	88659853	NM_004329	c.500T>C	p.(Met167Thr)	NA	NA	NA	0,000625	0,000313	NA

BRCA1	17	41243776	NM_007294	c.3770_3771delAG	p.(Glu1257Glyfs*9)	rs80357579	VCV000037546.66	NA	0,000625	0,000313	NA
BRCA1	17	41246692	NM_007294	c.856G>C	p.(Glu286Gln)	NA	NA	NA	0,000625	0,000313	NA
BRCA1	17	41246684	NM_007294	c.864C>A	p.(Ser288Arg)	NA	NA	NA	0,000625	0,000313	NA
BTD	3	15686731	ENST00000303498	c.1368A>C	p.(Gln456His)	rs80338685	VCV000001902.75	NA	0,000625	0,000313	4,00E-04
BTD	3	15686976	ENST00000303498	c.1613G>A	p.(Arg538His)	rs397514429	VCV000025099.14	NA	0,000625	0,000313	NA
BTD	3	15685832	ENST00000303498	c.469C>T	p.(Arg157Cys)	rs397514363	VCV000025012.11	NA	0,000625	0,000313	NA
BTD	3	15686732	ENST00000303498	c.1369G>A	p.(Val457Met)	rs146600671	VCV002203322.2	NA	0,000625	0,000313	NA
BTD	3	15685833	ENST00000303498	c.470G>A	p.(Arg157His)	rs146015592	VCV000038290.24	0.001	0,001875	0,000938	1,00E-04
BTD	3	15686958	ENST00000303498	c.1595C>T	p.(Thr532Met)	rs104893688	VCV000001897.45	NA	0,000625	0,000313	1,00E-04
BTD	3	15686112	ENST00000303498	c.749T>A	p.(Phe250Tyr)	NA	NA	NA	0,00125	0,000625	NA
BTD	3	15686813	ENST00000303498	c.1450T>C	p.(Phe484Leu)	NA	NA	NA	0,00125	0,000625	NA
BTD	3	15686156	ENST00000303498	c.793C>T	p.(His265Tyr)	NA	NA	NA	0,000625	0,000313	NA
BTD	3	15676990	ENST00000303498	c.104G>C	p.(Cys35Ser)	NA	NA	NA	0,001875	0,000938	NA
CACNA1S	1	201052427	NM_000069	c.1256G>T	p.(Arg419Leu)	NA	NA	NA	0,000625	0,000313	NA
CACNA1S	1	201054627	NM_000069	c.1087C>A	p.(Leu363Ile)	NA	NA	NA	0,000625	0,000313	NA
CACNA1S	1	201046193	NM_000069	c.1682C>T	p.(Ser561Phe)	NA	NA	NA	0,000625	0,000313	NA
CACNA1S	1	201036040	NM_000069	c.2632G>C	p.(Val878Leu)	NA	NA	NA	0,000625	0,000313	NA
CACNA1S	1	201029935	NM_000069	c.3265G>A	p.(Val1089Ile)	NA	NA	NA	0,000625	0,000313	NA
CACNA1S	1	201030492	NM_000069	c.3158C>A	p.(Ala1053Asp)	NA	NA	NA	0,000625	0,000313	NA
CACNA1S	1	201058414	NM_000069	c.872T>C	p.(Met291Thr)	NA	NA	NA	0,000625	0,000313	1,00E-04
CACNA1S	1	201057048	NM_000069	c.910G>A	p.(Ala304Thr)	NA	NA	NA	0,000625	0,000313	NA
CASQ2	1	116275527	NM_001232	c.601A>G	p.(Lys201Glu)	NA	NA	NA	0,000625	0,000313	NA
COL3A1	2	189875438	NM_000090	c.4076T>C	p.(Leu1359Pro)	NA	NA	NA	0,000625	0,000313	NA
COL3A1	2	189868849	NM_000090	c.2803C>A	p.(Pro935Thr)	NA	NA	NA	0,000625	0,000313	NA
DES	2	220290396	NM_001927	c.1300G>C	p.(Glu434Gln)	NA	NA	NA	0,001875	0,000938	NA
DES	2	220288519	NM_001927	c.1265C>T	p.(Thr422Ile)	NA	NA	NA	0,00125	0,000625	NA
DSC2	18	28651612	NM_004949	c.2084G>A	p.(Trp695*)	NA	NA	NA	0,000625	0,000313	NA
DSC2	18	28669467	NM_004949	c.565G>A	p.(Glu189Lys)	NA	NA	NA	0,000625	0,000313	NA
DSC2	18	28650817	NM_004949	c.2126-1G>C	p.?	NA	NA	NA	0,000625	0,000313	NA
DSG2	18	29125698	NM_001943	c.2349C>A	p.(Tyr783*)	rs1064793983	VCV000419603.5	NA	0,000625	0,000313	NA

DSG2	18	29102207	NM_001943	c.685A>G	p.(Arg229Gly)	NA	NA	NA	0,000625	0,000313	NA
DSP	6	7572210	NM_001008844	c.2039A>G	p.(Glu680Gly)	NA	NA	NA	0,000625	0,000313	NA
DSP	6	7580168	NM_001319034	c.3745C>G	p.(Leu1249Val)	NA	NA	NA	0,000625	0,000313	NA
DSP	6	7585057	NM_001008844	c.5765A>T	p.Asp1922Val	NA	NA	NA	0,000625	0,000313	NA
DSP	6	7583529	NM_001008844	c.4237C>T	p.(Leu1413Phe)	NA	NA	NA	0,000625	0,000313	NA
ENG	9	130587628	NM_000118	c.698C>A	p.(Thr233Lys)	NA	NA	NA	0,000625	0,000313	NA
FBN1	15	48782210	NM_000138	c.2920C>T	p.(Arg974Cys)	rs397514558	VCV000039667.28	NA	0,000625	0,000313	NA
FBN1	15	48760712	NM_000138	c.4479T>G	p.(Asp1493Glu)	NA	NA	NA	0,000625	0,000313	NA
FBN1	15	48780375	NM_000138	c.3272G>C	p.Gly1091Ala	NA	NA	NA	0,00125	0,000625	NA
FLNC	7	128483311	NM_001127487	c.2579A>G	p.(Lys860Arg)	NA	NA	NA	0,000625	0,000313	NA
FLNC	7	128486214	NM_001127487	c.3961G>C	p.(Glu1321Gln)	NA	NA	NA	0,000625	0,000313	NA
FLNC	7	128486950	NM_001127487	c.4279C>T	p.(Pro1427Ser)	NA	NA	NA	0,000625	0,000313	NA
FLNC	7	128478701	NM_001127487	c.1255G>C	p.(Gly419Arg)	NA	NA	NA	0,000625	0,000313	NA
FLNC	7	128498233	NM_001127487	c.7853G>T	p.(Gly2618Val)	NA	NA	NA	0,000625	0,000313	NA
GAA	17	78087081	NM_000152	c.2105G>A	p.(Arg702His)	rs398123172	VCV000426278.28	NA	0,000625	0,000313	1,00E-04
GAA	17	78078341	NM_000152	c.-32-13T>C	p.(?)	rs386834236	VCV001025528.5	NA	0,005625	0,00125	0,0035
GAA	17	78084737	NM_000152	c.1552-3C>G	p.(?)	rs375470378	VCV000419722.50	NA	0,00125	0,000625	1,00E-04
GAA	17	78086797	NM_000152	c.2011A>G	p.(Met671Val)	NA	NA	NA	0,000625	0,000313	NA
GAA	17	78091528	NM_000152	c.2461G>T	p.(Gly821Trp)	NA	NA	NA	0,00125	0,000625	NA
GAA	17	78078655	NM_000152	c.270C>G	p.(Phe90Leu)	NA	NA	NA	0,000625	0,000313	NA
HFE	6	26091185	NM_000410	c.193A>T	p.(Ser65Cys)	rs1800730	VCV000000011.75	0,006	0,005625	0,002813	0,0102
HFE	6	26093141	NM_000410	c.845G>A	p.(Cys282Tyr)	rs1800562	VCV000000009.115	0,004	0,01	0,005313	0,0332
HFE	6	26091179	NM_000410	c.187C>G	p.(His63Asp)	rs1799945	VCV000000010.111	0,093	0,2275	0,120938	0,1095
HNF1A	12	121434371	NM_000545	c.1135C>T	p.(Pro379Ser)	rs754729248	VCV002052397.3	NA	0,00125	0,000625	NA
KCNH2	7	150655153	NM_000238	c.910A>C	p.(Ser304Arg)	NA	NA	NA	0,000625	0,000313	NA
KCNQ1	11	2594209	NM_000218	c.914G>A	p.(Trp305*)	rs120074186	VCV000053124.25	NA	0,000625	0,000313	NA
KCNQ1	11	2549232	NM_000218	c.461G>A	p.(Gly154Glu)	NA	NA	NA	0,000625	0,000625	NA
KCNQ1	11	2790105	NM_000218	c.1546G>A	p.(Val516Ile)	NA	NA	NA	0,000625	0,000625	NA
LDLR	19	11218108	NM_000527	c.858C>A	p.(Ser286Arg)	rs140241383	VCV000251488.49	NA	0,000625	0,000313	NA
LDLR	19	11230767	NM_000527	c.1846-1G>A	p.(?)	rs879255051	VCV000252079.21	NA	0,000625	0,000313	NA

LMNA	1	156084738	NM_001282625	c.29C>T	p.(Thr10Ile)	rs57077886	VCV000066888.10	NA	0,000625	0,000313	NA
LMNA	1	156107508	NM_001257374	c.1336G>A	p.(Gly446Arg)	NA	NA	NA	0,000625	0,000313	NA
MSH2	2	47709953	NM_000251	c.2670G>T	p.(Lys890Asn)	NA	NA	NA	0,00125	0,000625	NA
MUTYH	1	45797348	ENST00000528013	c.1129C>T	p.(Gln377*)	rs587783057	VCV000156509.45	NA	0,000625	0,000313	NA
MUTYH	1	45797228	ENST00000528013	c.1145G>A	p.(Gly382Asp)	rs36053993	VCV000645786.7	NA	0,0025	0,00125	0,0031
MUTYH	1	45798117	ENST00000528013	c.692G>A	p.(Arg231His)	rs140342925	VCV000140877.59	NA	0,00125	0,000625	1,00E-04
MUTYH	1	45799121	ENST00000528013	c.270C>A	p.(Tyr90*)	rs121908380	VCV000005296.52	NA	0,00125	0,000625	1,00E-04
MUTYH	1	45797887	ENST00000528013	c.842C>T	p.(Pro281Leu)	rs374950566	VCV000185242.62	0,004	0,005625	0,002813	NA
MUTYH	1	45796905	ENST00000528013	c.1383G>T	p.(Trp461Cys)	NA	NA	NA	0,000625	0,000313	NA
MYBPC3	11	47364270	NM_000256	c.1483C>T	p.(Arg495Trp)	rs397515905	VCV000164114.29	NA	0,000625	0,000313	NA
MYBPC3	11	47356671	NM_000256	c.2827C>T	p.(Arg943*)	rs387907267	VCV000037039.40	NA	0,000625	0,000313	NA
MYBPC3	11	47354525	NM_000256	c.3331-1G>A	p.?	rs727504305	VCV000177742.12	NA	0,000625	0,000625	NA
MYBPC3	11	47359310	NM_000256	c.2344A>T	p.(Asn782Tyr)	NA	NA	NA	0,000625	0,000313	NA
MYH11	16	15850362	NM_001040113	c.1606C>G	p.(Pro536Ala)	NA	NA	NA	0,000625	0,000313	NA
MYH11	16	15841904	NM_001040113	c.2201G>A	p.(Arg734His)	NA	NA	NA	0,000625	0,000313	NA
MYH11	16	15917169	NM_001040113	c.445G>A	p.(Glu149Lys)	NA	NA	NA	0,000625	0,000313	NA
MYH11	16	15917192	NM_001040113	c.422T>C	p.(Met141Thr)	NA	NA	NA	0,00125	0,000625	NA
MYH11	16	15802700	NM_001040113	c.5808-2A>C	p.?	NA	NA	NA	0,000625	0,000313	NA
MYH7	14	23895201	NM_000257	c.2134C>T	p.(Arg712Cys)	rs749007293	VCV000407185.11	NA	0,00125	0,000625	NA
MYH7	14	23894051	NM_000257	c.2606G>A	p.(Arg869His)	rs202141173	VCV000177667.70	NA	0,000625	0,000313	NA
MYH7	14	23895189	NM_000257	c.2146G>A	p.(Gly716Arg)	rs121913638	VCV000014105.22	NA	0,000625	0,000313	NA
MYH7	14	23901862	NM_000257	c.488A>C	p.(Gln163Pro)	NA	VCV001879272.14	NA	0,000625	0,000313	NA
MYH7	14	23897042	NM_000257	c.1640C>T	p.(Thr547Ile	NA	VCV002498591.12	NA	0,000625	0,000313	NA
MYH7	14	23886525	NM_000257	c.4356C>G	p.(Ile1452Met)	NA	VCV003074372.1	NA	0,000625	0,000313	NA
MYL3	3	46901001	NM_000258	c.445A>G	p.(Met149Val)	rs104893748	VCV000014061.6	NA	0,000625	0,000313	NA
MYL3	3	46902291	NM_000258	c.182T>C	p.(Phe61Ser)	NA	NA	NA	0,000625	0,000313	NA
PALB2	16	23641218	NM_024675	c.2257C>T	p.(Arg753*)	rs180177110	VCV000142403.81	NA	0,000625	0,000313	NA
PALB2	16	23646617	NM_024675	c.1250C>T	p.(Ser417Phe)	NA	NA	NA	0,000625	0,000313	NA
PCSK9	1	55523883	NM_174936	c.1354+1G>A	p.?	NA	NA	NA	0,000625	0,000313	NA
PKP2	12	32974418	NM_001005242	c.1885G>A	p.(Gly629Ser)	NA	NA	NA	0,001875	0,000938	NA

PKP2	12	33031068	NM_001005242	c.746G>C	p.(Ser249Thr)	NA	NA	NA	0,000625	0,000313	NA
PMS2	7	6017343	NM_000535	c.2321A>T	p.(Lys774Ile)	NA	NA	NA	0,000625	0,000313	NA
PRKAG2	7	151372667	NM_001040633	c.391T>C	p.(Phe131Leu)	NA	NA	NA	0,000625	0,000313	NA
RBM20	10	112541286	NM_001134363	c.919G>T	p.(Glu307*)	NA	NA	NA	0,000625	0,000313	NA
RBM20	10	112540708	NM_001134363	c.341C>T	p.(Thr114Ile)	NA	NA	NA	0,000625	0,000313	NA
RBM20	10	112541157	NM_001134363	c.790G>A	p.(Glu264Lys)	NA	NA	NA	0,000625	0,000313	NA
RET	10	43612152	NM_020630	c.2257A>C	p.(Thr753Pro)	NA	NA	NA	0,000625	0,000313	NA
RPE65	1	68910508	NM_000329	c.304G>T	p.(Glu102*)	rs62642584	VCV000098863.43	NA	0,000625	0,000313	NA
RPE65	1	68910339	NM_000329	c.370C>T	p.(Arg124*)	rs61752877	VCV000098866.23	NA	0,000625	0,000313	NA
RPE65	1	68897237	NM_000329	c.1160C>G	p.(Pro387Arg)	NA	NA	NA	0,00125	0,000625	NA
RPE65	1	68906566	NM_000329	c.613A>C	p.(Asn205His)	NA	NA	NA	0,00125	0,000625	NA
RPE65	1	68914330	NM_000329	c.71C>T	p.(Ser24Leu)	NA	NA	NA	0,000625	0,000313	NA
RPE65	1	68896765	NM_000329	c.1433C>G	p.(Ala478Gly)	NA	NA	NA	0,000625	0,000313	NA
RYR1	19	39013751	NM_000540	c.10343C>T	p.(Ser3448Phe)	rs193922836	VCV000012991.4	NA	0,00125	0,000625	NA
RYR1	19	39077216	NM_000540	c.15021G>C	p.(Gln5007His)	NA	NA	NA	0,000625	0,000313	NA
RYR1	19	38987526	NM_000540	c.6823G>A	p.(Val2275Met)	NA	NA	NA	0,000625	0,000313	NA
RYR1	19	38939032	NM_000540	c.838C>T	p.(Arg280*)	NA	NA	NA	0,000625	0,000313	NA
RYR1	19	39019322	NM_000540	c.11021T>C	p.(Ile3674Thr)	NA	NA	NA	0,00125	0,000625	NA
RYR1	19	39055647	NM_000540	c.12673G>A	p.(Gly4225Ser)	NA	NA	NA	0,000625	0,000313	NA
RYR1	19	38997471	NM_000540	c.8695G>A	p.(Gly2899Ser)	NA	NA	NA	0,000625	0,000313	NA
RYR1	19	39034189	NM_000540	c.11796C>G	p.(Phe3932Leu)	NA	NA	NA	0,000625	0,000313	NA
RYR1	19	38948245	NM_000540	c.1900A>G	p.(Thr634Ala)	NA	NA	NA	0,000625	0,000313	NA
RYR1	19	38943602	NM_000540	c.1388A>C	p.(Lys463Thr)	NA	NA	NA	0,000625	0,000313	NA
RYR1	19	38996563	NM_000540	c.8518C>G	p.(Arg2840Gly)	NA	NA	NA	0,00125	0,000625	NA
RYR1	19	39019297	NM_000540	c.10996G>A	p.(Asp3666Asn)	NA	NA	NA	0,000625	0,000313	NA
RYR1	19	38993361	NM_000540	c.7829T>C	p.(Leu2610Pro)	NA	NA	NA	0,000625	0,000313	NA
RYR1	19	38968483	NM_000540	c.4427G>A	p.(Gly1476Glu)	NA	NA	NA	0,00125	0,000625	NA
RYR1	19	39052071	NM_000540	c.12601C>A	p.(Arg4201Ser)	NA	NA	NA	0,000625	0,000313	NA
RYR1	19	38976786	NM_000540	c.5491G>A	p.(Gly1831Arg)	NA	NA	NA	0,000625	0,000313	NA
RYR1	19	38995473	NM_000540	c.8153C>T	p.(Ser2718Leu)	NA	NA	NA	0,000625	0,000313	NA

RYR1	19	38956961	NM_000540	c.3101A>G	p.(Asn1034Ser)	NA	NA	NA	0,000625	0,000313	NA
RYR1	19	38983211	NM_000540	c.6209T>A	p.(Val2070Glu)	NA	NA	NA	0,000625	0,000313	NA
RYR1	19	39025835	NM_000540	c.11414A>G	p.(Asn3805Ser)	NA	NA	0,003	0,00125	0,000625	NA
RYR1	19	38989770	NM_000540	c.6914G>C	p.(Cys2305Ser)	NA	NA	NA	0,000625	0,000313	NA
RYR1	19	38991586	NM_000540	c.7570G>A	p.(Val2524Met)	NA	NA	NA	0,000625	0,000313	NA
RYR1	19	39063946	NM_000540	c.14128C>G	p.(Pro4710Ala)	NA	NA	NA	0,000625	0,000313	NA
RYR2	1	237753294	NM_001035	c.3800A>T	p.(His1267Leu)	NA	NA	NA	0,001875	0,000938	NA
RYR2	1	237713852	NM_001035	c.3075G>T	p.(Lys1025Asn)	NA	NA	NA	0,000625	0,000313	NA
RYR2	1	237837516	NM_001035	c.8711C>T	p.(Ser2904Phe)	NA	NA	NA	0,000625	0,000313	NA
RYR2	1	237604738	NM_001035	c.1125G>C	p.(Gln375His)	NA	NA	NA	0,000625	0,000313	NA
RYR2	1	237586483	NM_001035	c.940C>T	p.(Leu314Phe)	NA	NA	NA	0,000625	0,000313	NA
RYR2	1	237551452	NM_001035	c.742C>T	p.(Pro248Ser)	NA	NA	NA	0,000625	0,000313	NA
SCN5A	3	38593010	NM_000335	c.4847_4849del	p.(Phe1616del)	rs749697698	VCV000201572.31	NA	0,000625	0,000313	NA
SCN5A	3	38601775	NM_000335	c.4105G>T	p.(Asp1369Tyr)	NA	NA	NA	0,000625	0,000313	NA
SCN5A	3	38593008	NM_000335	c.4852C>G	p.(Pro1618Ala)	NA	NA	NA	0,000625	0,000313	NA
SCN5A	3	38627384	NM_000335	c.2585G>C	p.(Gly862Ala)	NA	NA	NA	0,000625	0,000313	NA
SCN5A	3	38591823	NM_000335	c.6037T>A	p.(Ser2013Thr)	NA	NA	NA	0,000625	0,000313	NA
SCN5A	3	38622540	NM_000335	c.3110G>T	p.(Gly1037Val)	NA	NA	NA	0,000625	0,000313	NA
SCN5A	3	38607943	NM_000335	c.3794A>G	p.(Tyr1265Cys)	NA	NA	NA	0,000625	0,000313	NA
SDHB	1	17371313	NM_003000	c.143A>T	p.(Asp48Val)	rs202101384	VCV000039584.40	NA	0,000625	0,000313	NA
TGFBR2	3	30732970	NM_001024847	c.1658G>A	p.(Arg553His)	rs104893815	VCV000012511.11	NA	0,000625	0,000313	NA
TGFBR2	3	30648410	NM_001024847	c.35T>A	p.(Leu12Gln)	NA	NA	NA	0,000625	0,000313	NA
TMEM43	3	14172350	NM_024334	c.191T>G	p.(Leu64Trp)	NA	NA	NA	0,000625	0,000313	NA
TNNT2	1	201330484	NM_000364	c.724G>A	p.(Glu242Lys)	NA	NA	NA	0,00125	0,000625	NA
TSC2	16	2130180	NM_000548	c.3412C>T	p.(Arg1138*)	rs45451497	VCV000049257.38	NA	0,000625	0,000313	NA
TSC2	16	2115563	NM_000548	c.1643G>A	p.(Arg548Lys)	NA	NA	NA	0,000625	0,000313	NA
TSC2	16	2126157	NM_000548	c.2728C>G	p.(Pro910Ala)	NA	NA	NA	0,000625	0,000313	NA
TTN	2	179530520	NM_001267550	c.35876-2A>G	p.?	NA	VCV000809024.26	NA	0,00125	0,000625	NA
TTN	2	179457643	NM_001256850	c.54278_54279delCT	p.(Pro18093Argfs*5)	rs752948913	VCV000419310.15	NA	0,000625	0,000313	NA
TTN	2	179585309	NM_001256850	c.22229G>A	p.(Gly7410Glu)	rs763907919	VCV002651685.8	NA	0,000625	0,000625	NA

TTN	2	179641568	NM_001256850	c.5023C>T	p.(Pro1675Ser)	NA	NA	NA	0,00125	0,00125	NA
TTN	2	179616188	NM_133379	c.10939G>T	p.(Asp3647Tyr)	NA	NA	NA	0,000625	0,000313	NA
TTN	2	179659257	NM_001256850	c.1267A>C	p.(Ser423Arg)	NA	NA	NA	0,00125	0,000625	NA
TTN	2	179571302	NM_001256850	c.28348G>C	p.(Gly9450Arg)	NA	NA	NA	0,000625	0,000313	NA
TTN	2	179435741	NM_001256850	c.70195G>A	p.(Gly23399Ser)	NA	NA	NA	0,00125	0,000625	NA
TTN	2	179471823	NM_001256850	c.48583C>T	p.(Arg16195Cys)	NA	NA	NA	0,000625	0,000313	NA
TTN	2	179462764	NM_001256850	c.52210G>C	p.(Gly17404Arg)	NA	NA	NA	0,000625	0,000313	NA
TTN	2	179635943	NM_001256850	c.8111T>C	p.(Val2704Ala)	NA	NA	NA	0,000625	0,000313	NA
TTN	2	179416711	NM_001256850	c.85993G>A	p.(Gly28665Arg)	NA	NA	NA	0,00125	0,000625	NA
TTN	2	179638934	NM_001256850	c.7057C>T	p.(Pro2353Ser)	NA	NA	NA	0,00125	0,000625	NA
TTN	2	179432742	NM_001256850	c.73194G>C	p.(Lys24398Asn)	NA	NA	NA	0,000625	0,000313	NA
TTN	2	179640824	NM_001256850	c.5767C>T	p.(His1923Tyr)	NA	NA	NA	0,000625	0,000313	NA
TTN	2	179393027	NM_001256850	c.102428C>T	p.(Ser34143Leu)	NA	NA	NA	0,000625	0,000313	NA
TTN	2	179397484	NM_001256850	c.98935C>T	p.(Arg32979Cys)	NA	NA	NA	0,000625	0,000313	NA
TTN	2	179457978	NM_001256850	c.54034C>T	p.(Arg18012Trp)	NA	NA	NA	0,000625	0,000313	NA
TTN	2	179667068	NM_001256850	c.92G>A	p.(Gly31Asp)	NA	NA	NA	0,000625	0,000313	NA
TTN	2	179486663	NM_001256850	c.40063C>A	p.(Arg13355Ser)	NA	NA	NA	0,000625	0,000313	NA
TTN	2	179473615	NM_001256850	c.47200A>T	p.(Asn15734Tyr)	NA	NA	NA	0,00125	0,000625	NA
TTN	2	179393025	NM_001256850	c.102430G>C	p.(Ala34144Pro)	NA	NA	NA	0,000625	0,000313	NA
TTN	2	179641213	NM_001256850	c.5378T>C	p.(Ile1793Thr)	NA	NA	NA	0,000625	0,000313	NA
TTN	2	179433151	NM_001256850	c.72785T>A	p.(Val24262Asp)	NA	NA	NA	0,00125	0,000625	NA
TTN	2	179577869	NM_001256850	c.26041T>G	p.(Cys8681Gly)	NA	NA	NA	0,00125	0,000625	NA
TTN	2	179403426	NM_001256850	c.94207G>C	p.(Ala31403Pro)	NA	NA	NA	0,000625	0,000313	NA
TTN	2	179438107	NM_001256850	c.67829G>A	p.(Gly22610Glu)	NA	NA	NA	0,000625	0,000313	NA
TTN	2	179443676	NM_001256850	c.63158G>A	p.(Cys21053Tyr)	NA	NA	NA	0,001875	0,000938	NA
TTN	2	179501464	NM_001256850	c.36067G>A	p.(Gly12023Ser)	NA	NA	NA	0,000625	0,000313	NA
TTN	2	179575512	NM_001256850	c.27361C>T	p.(Arg9121*)	NA	NA	NA	0,000625	0,000313	NA
TTN	2	179455970	NM_001256850	c.55559C>T	p.(Thr18520Ile)	NA	NA	NA	0,003125	0,001563	NA
TTN	2	179449122	NM_001256850	c.60233A>G	p.(Tyr20078Cys)	NA	NA	NA	0,00125	0,000625	NA
TTN	2	179442010	NM_001256850	c.64129T>C	p.(Tyr21377His)	NA	NA	NA	0,00125	0,000625	NA

TTN	2	179433406	NM_001256850	c.72530A>C	p.(Glu24177Ala)	NA	NA	0,003	0,000625	0,000313	NA
TTN	2	179456896	NM_001256850	c.54812A>G	p.(Tyr18271Cys)	NA	NA	NA	0,000625	0,000313	NA
TTN	2	179437292	NM_001256850	c.68644C>T	p.(Pro22882Ser)	NA	NA	NA	0,000625	0,000313	NA
TTN	2	179640215	NM_001256850	c.6376T>C	p.(Trp2126Arg)	NA	NA	NA	0,000625	0,000313	NA
TTN	2	179496027	NM_001256850	c.38825A>G	p.(Glu12942Gly)	NA	NA	NA	0,00125	0,000625	NA
TTN	2	179641452	NM_001256850	c.5139A>T	p.(Arg1713Ser)	NA	NA	NA	0,000625	0,000313	NA
TTN	2	179433083	NM_001256850	c.72853T>C	p.(Tyr24285His)	NA	NA	NA	0,000625	0,000313	NA
TTN	2	179443338	NM_001256850	c.63406G>A	p.(Gly21136Arg)	NA	NA	NA	0,000625	0,000313	NA
TTN	2	179455451	NM_001256850	c.56078G>A	p.(Gly18693Glu)	NA	NA	NA	0,000625	0,000313	NA
TTN	2	179458964	NM_001256850	c.53233C>G	p.(Pro17745Ala)	NA	NA	NA	0,000625	0,000313	NA
TTN	2	179457657	NM_001256850	c.54266C>A	p.(Ala18089Asp)	NA	NA	NA	0,000625	0,000313	NA
TTN	2	179442021	NM_001256850	c.64118A>G	p.(Asp21373Gly)	NA	NA	NA	0,00125	0,000625	NA
TTN	2	179424589	NM_001256850	c.81347A>C	p.(Asp27116Ala)	NA	NA	NA	0,000625	0,000313	NA
TTN	2	179431708	NM_001256850	c.74228T>C	p.(Phe24743Ser)	NA	NA	NA	0,000625	0,000313	NA
TTN	2	179640323	NM_001256850	c.6268G>C	p.(Gly2090Arg)	NA	NA	NA	0,000625	0,000313	NA
TTN	2	179418022	NM_001256850	c.84682C>T	p.(Pro28228Ser)	NA	NA	NA	0,000625	0,000313	NA
TTN	2	179614594	NM_133379	c.12533T>C	p.(Leu4178Pro)	NA	NA	NA	0,00125	0,000625	NA
TTN	2	179610888	NM_133379	c.16239G>T	p.(Trp5413Cys)	NA	NA	NA	0,001875	0,000938	NA
TTN	2	179400877	NM_001256850	c.95674G>A	p.(Gly31892Ser)	NA	NA	NA	0,00125	0,000625	NA
TTN	2	179435831	NM_001256850	c.70105C>T	p.(Pro23369Ser)	NA	NA	NA	0,000625	0,000313	NA
TTN	2	179664253	NM_001256850	c.875C>T	p.(Pro292Leu)	NA	NA	NA	0,00125	0,000625	NA
TTN	2	179639840	NM_001256850	c.6598G>A	p.(Glu2200Lys)	NA	NA	NA	0,000625	0,000313	NA
TTN	2	179431859	NM_001256850	c.74077T>C	p.(Trp24693Arg)	NA	NA	NA	0,00125	0,000625	NA
TTN	2	179497744	NM_001256850	c.38191A>C	p.(Lys12731Gln)	NA	NA	NA	0,000625	0,000313	NA
TTN	2	179434567	NM_001256850	c.71369A>G	p.(Tyr23790Cys)	NA	NA	NA	0,000625	0,000313	NA
TTN	2	179600306	NM_001256850	c.13916G>A	p.(Gly4639Glu)	NA	NA	NA	0,000625	0,000313	NA
TTN	2	179640896	NM_001256850	c.5695A>T	p.(Ile1899Phe)	NA	NA	NA	0,000625	0,000313	NA
TTN	2	179458924	NM_001256850	c.53273G>C	p.Arg17758Pro	NA	NA	NA	0,000625	0,000313	NA
TTN	2	179428384	NM_001256850	c.77552G>A	p.(Arg25851His)	NA	NA	NA	0,00125	0,000625	NA
TTN	2	179404834	NM_001256850	c.93136G>C	p.(Ala31046Pro)	NA	NA	NA	0,000625	0,000313	NA

TTN	2	179422158	NM_001256850	c.82908A>C	p.(Lys27636Asn)	NA	NA	NA	0,00125	0,000625	NA
TTN	2	179665145	NM_001256850	c.560C>T	p.(Ser187Leu)	NA	NA	NA	0,000625	0,000313	NA
TTN	2	179632834	NM_001256850	c.9212A>G	p.(Glu3071Gly)	NA	NA	NA	0,00125	0,000625	NA
TTN	2	179456803	NM_001256850	c.54905A>G	p.(Glu18302Gly)	NA	NA	NA	0,000625	0,000313	NA
TTN	2	179437239	NM_001256850	c.68697G>T	p.(Trp22899Cys)	NA	NA	NA	0,000625	0,000313	NA
TTN	2	179458918	NM_001256850	c.53279G>C	p.(Gly17760Ala)	NA	NA	NA	0,000625	0,000313	NA
TTN	2	179430433	NM_001256850	c.75503G>A	p.(Gly25168Asp)	NA	NA	NA	0,000625	0,000313	NA
TTN	2	179407278	NM_001256850	c.92282C>G	p.(Pro30761Arg)	NA	NA	0,002	0,000625	0,000313	NA
TTN	2	179411849	NM_001256850	c.89480A>G	p.(Tyr29827Cys)	NA	NA	NA	0,000625	0,000313	NA
TTN	2	179443547	NM_001256850	c.63287C>T	p.(Ala21096Val)	NA	NA	NA	0,000625	0,000313	NA
TTN	2	179454744	NM_001256850	c.56785C>G	p.(Pro18929Ala)	NA	NA	NA	0,000625	0,000313	NA
TTN	2	179567202	NM_001256850	c.29461A>C	p.(Asn9821His)	NA	NA	NA	0,000625	0,000313	NA
TTN	2	179455958	NM_001256850	c.55571A>G	p.(His18524Arg)	NA	NA	NA	0,000625	0,000313	1,00E-04
TTN	2	179664340	NM_001256850	c.788C>A	p.(Ser263*)	NA	NA	NA	0,000625	0,000313	NA
TTN	2	179442138	NM_001256850	c.64001C>A	p.(Pro21334His)	NA	NA	NA	0,000625	0,000313	NA
TTN	2	179440532	NM_001256850	c.65404C>T	p.(Arg21802Trp)	NA	NA	NA	0,00125	0,000625	NA
TTN	2	179463724	NM_001256850	c.51790T>A	p.(Trp17264Arg)	NA	NA	NA	0,000625	0,000313	NA
TTN	2	179429593	NM_001256850	c.76343T>G	p.(Met25448Arg)	NA	NA	NA	0,000625	0,000313	NA
TTN	2	179446526	NM_001256850	c.61546C>T	p.(Pro20516Ser)	NA	NA	NA	0,000625	0,000313	NA
TTN	2	179438708	NM_001256850	c.67228G>A	p.(Ala22410Thr)	NA	NA	NA	0,000625	0,000313	NA
TTN	2	179446708	NM_001256850	c.61465G>C	p.(Val20489Leu)	NA	NA	NA	0,000625	0,000313	NA
TTN	2	179458553	NM_001256850	c.53551G>A	p.(Val17851Met)	NA	NA	NA	0,000625	0,000313	NA
TTN	2	179402096	NM_001256850	c.94915G>C	p.(Asp31639His)	NA	NA	NA	0,000625	0,000313	NA
TTN	2	179606105	NM_001256850	c.10904G>T	p.(Gly3635Val)	NA	NA	NA	0,000625	0,000313	NA
TTN	2	179489431	NM_001256850	c.39653T>C	p.(Leu13218Pro)	NA	NA	NA	0,000625	0,000313	NA
TTN	2	179464163	NM_001256850	c.51434G>A	p.Gly17145Asp	NA	NA	NA	0,000625	0,000313	NA
TTN	2	179433316	NM_001256850	c.72620A>T	p.(Asp24207Val)	NA	NA	NA	0,000625	0,000313	NA
TTN	2	179458337	NM_001256850	c.53767G>T	p.(Asp17923Tyr)	NA	NA	NA	0,000625	0,000313	NA
TTN	2	179401917	NM_001256850	c.94996T>C	p.(Ser31666Pro)	NA	NA	NA	0,000625	0,000313	NA
TTN	2	179513976	NM_001267550	c.40056A>C	p.(Lys13352Asn)	NA	NA	NA	0,000625	0,000313	NA

TTN	2	179404450	NM_001256850	c.93419G>A	p.(Cys31140Tyr)	NA	NA	NA	0,000625	0,000313	NA
TTN	2	179482810	NM_001256850	c.42347-2A>G	p.?	NA	NA	NA	0,000625	0,000313	NA
TTR	18	29172931	NM_000371	c.142G>T	p.(Val48Leu)	NA	NA	NA	0,000625	0,000313	NA
VHL	3	10191506	NM_000551	c.499C>T	p.(Arg167Trp)	rs5030820	VCV000002219.11	NA	0,000625	0,000313	NA

AF: Alel frequency, **Freq:** Frequency, **LP:** Likely pathogenic, **NA:** Not available, **P:** Pathogenic, **LP:** Likely pathogenic, **TGPDSPTF:** Turkey Genome Project Data Sharing Portal frequency

Supplementary Figures

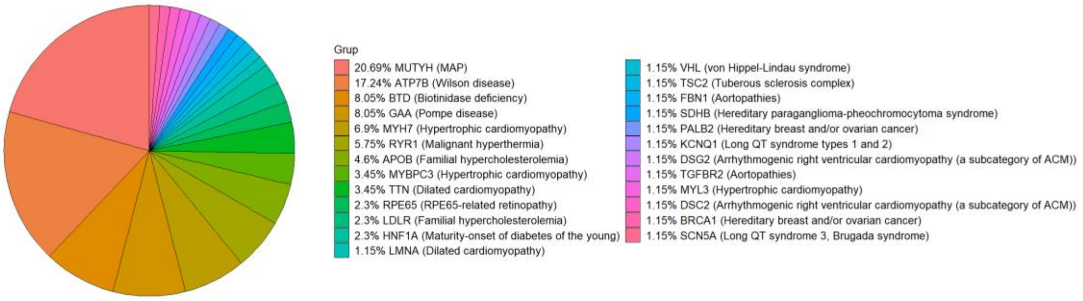

Supplementary Figure S1. Distribution of ACMG secondary findings by gene. The pie chart illustrates the proportion of pathogenic or likely pathogenic variants identified in the 81 ACMG-recommended genes among the study cohort.

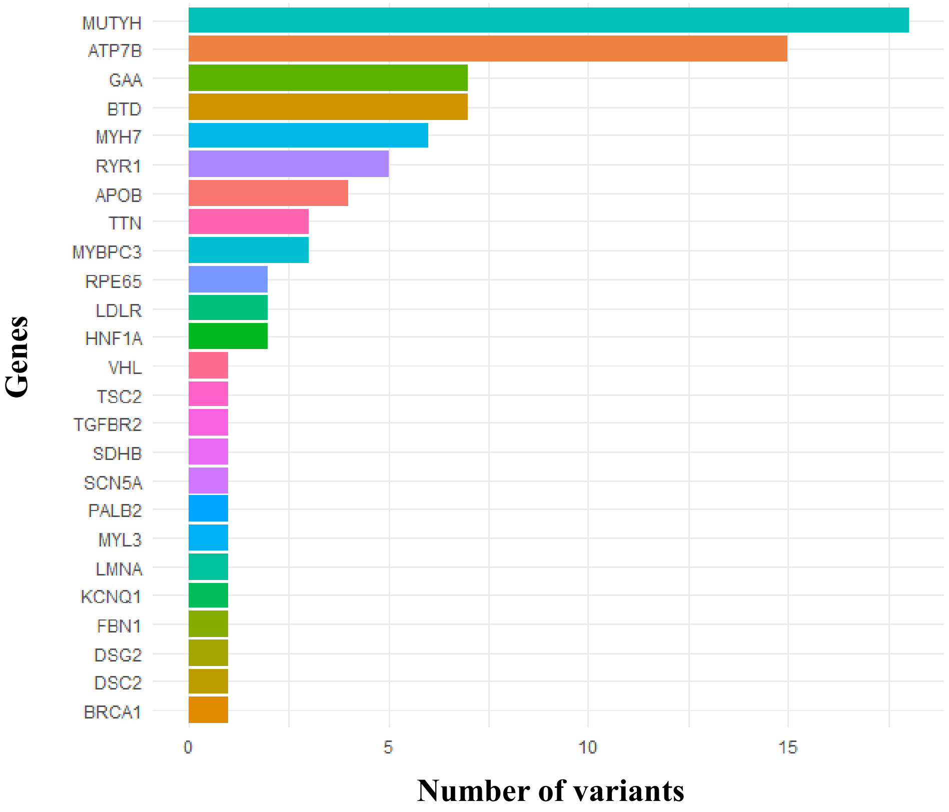

Supplementary Figure S2. Number of variants identified in genes reported as pathogenic/ likely pathogenic in ClinVar

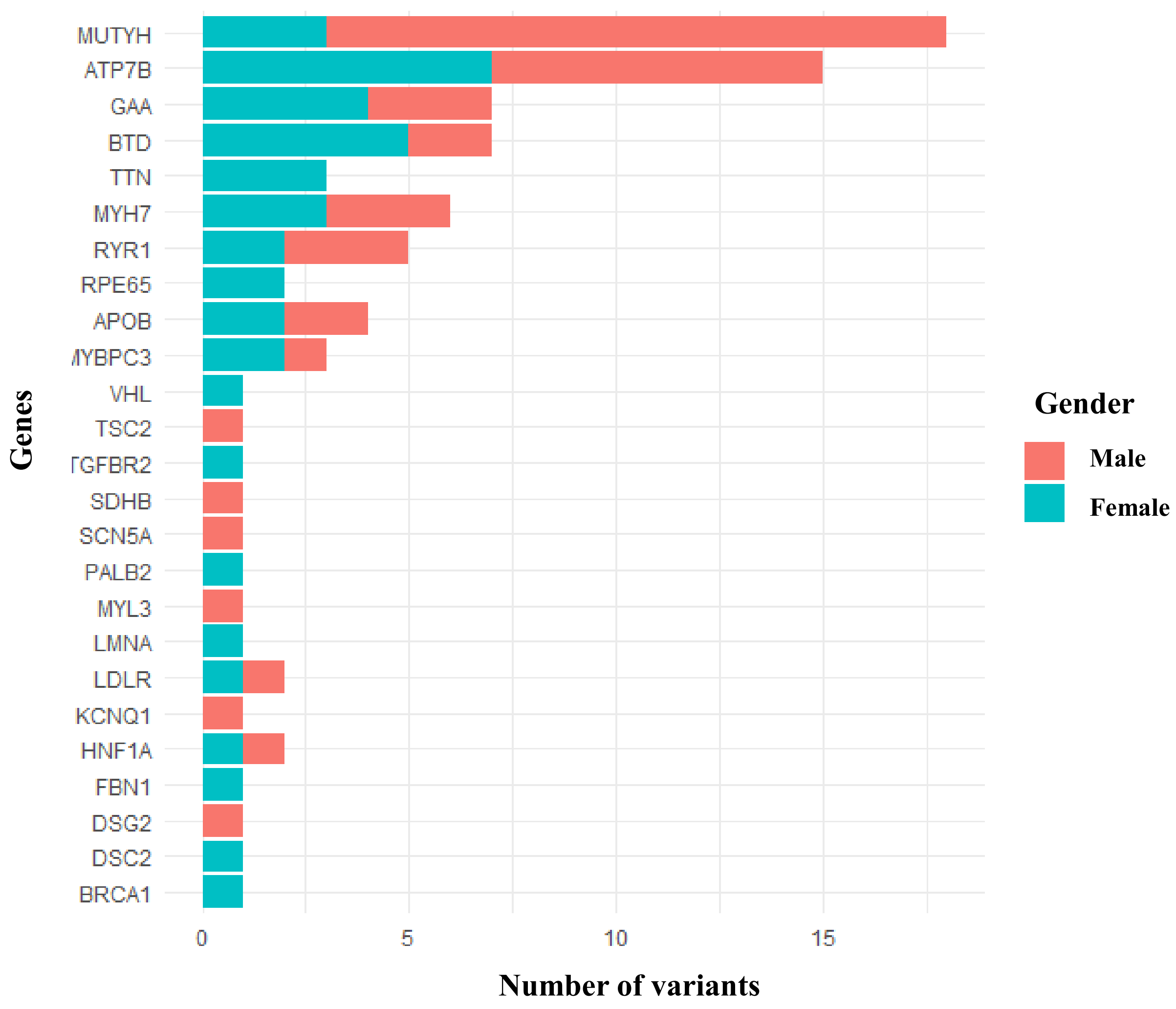

Supplementary Figure S3. Gender distribution of variants identified in genes reported as pathogenic/likely pathogenic in ClinVar

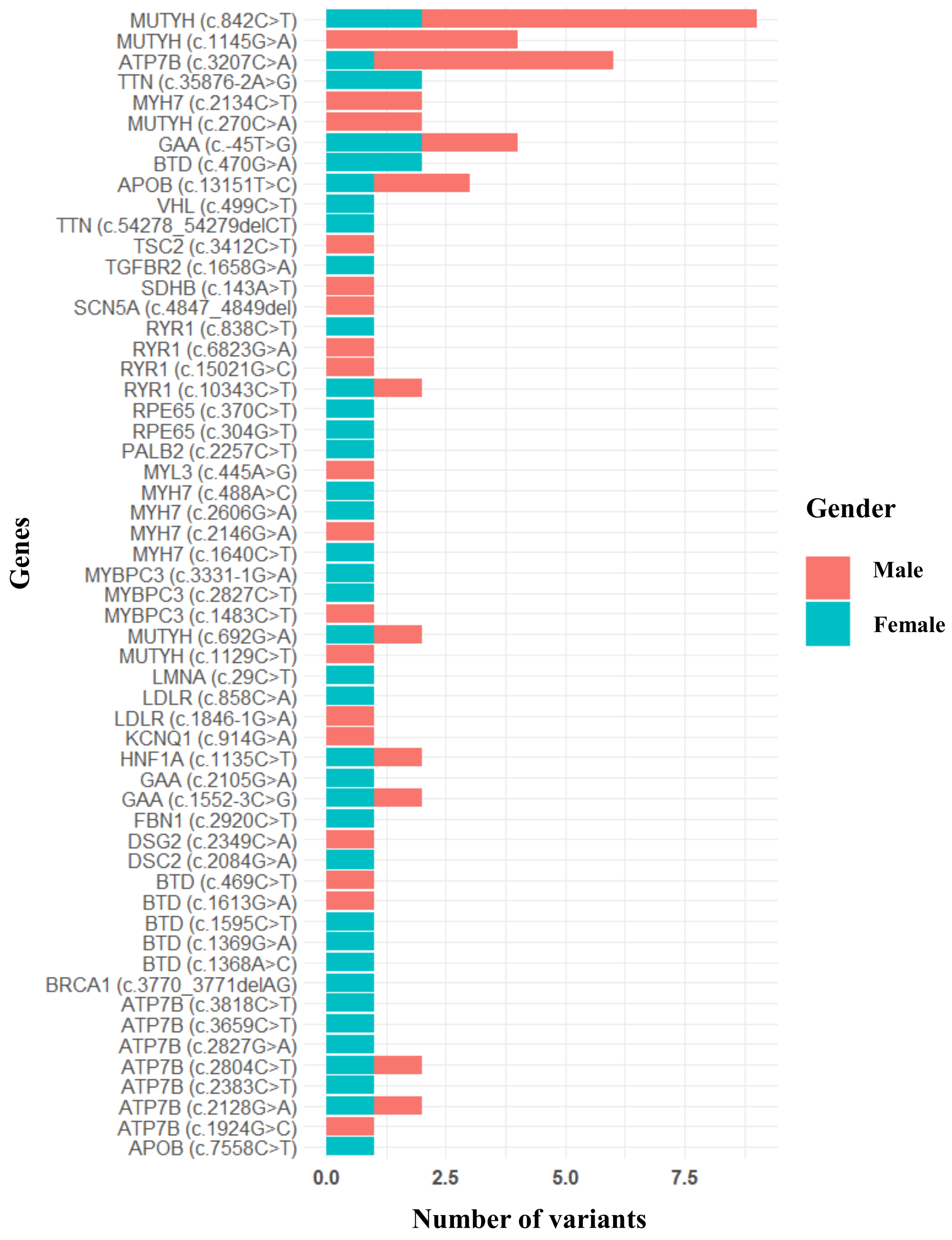

Supplementary Figure S4. Gender distribution of detected variants in genes reported as pathogenic/likely pathogenic in ClinVar
